# Preserving Nipple Sensitivity after Breast Cancer Surgery: A Systematic Review and Meta-Analysis

**DOI:** 10.1155/2022/9654741

**Published:** 2022-11-19

**Authors:** Varsha Harish, Zoe K. Haffner, Jenna C. Bekeny, Adaah A. Sayyed, David H. Song, Kenneth L. Fan

**Affiliations:** ^1^Georgetown University School of Medicine, Washington, District of Columbia, USA; ^2^Department of Plastic and Reconstructive Surgery, MedStar Georgetown University Hospital, Washington, District of Columbia, USA

## Abstract

**Purpose:**

As breast-conserving procedures become increasingly safe and viable options for surgical management of breast cancer, efforts have focused on assessing and optimizing patient-reported outcome measures (PROMs), such as nipple sensation. This study aims to evaluate the current understanding of nipple-areolar complex (NAC) sensation outcomes in breast cancer patients undergoing breast cancer surgeries, namely, nipple-sparing mastectomies (NSM), skin-sparing mastectomies (SSM), and lumpectomies.

**Methods:**

Articles including terms related to “nipple,” “mastectomy,” “sensation,” and “patient-reported outcome” were queried from three databases according to PRISMA guidelines. Study characteristics, patient demographics, and surgical details were recorded. Outcomes of interest included objective nipple sensitivity testing and PROMs.

**Results:**

Of 888 manuscripts identified, 28 articles met the inclusion criteria. Twelve studies (*n* = 578 patients) used objective measures to evaluate sensitivity, such as monofilament testing. Sixteen studies (*n* = 1785 patients) assessed PROMs through validated or investigator-generated surveys. Three of the included studies reported NAC sensitivity in patients who received NSM with neurotization (*n* = 203 patients) through a variety of techniques that used various grafts to coapt a lateral intercostal nerve to the NAC nerve stumps. Results of investigator surveys showed that of 1565 patients without neurotization, nipple sensation was maintained in 29.0% (*n* = 453) of patients. Of 138 NSM patients without NAC neurotization, SWM testing showed an average loss of protective sensation in the nipple (average SWM score: 4.7) compared to normal or diminished sensation to light touch in nonoperated controls (average SWM score: 2.9, *n* = 195). Of patients who underwent NSM with neurotization, one study (*n* = 78) reported maintenance of NAC sensation in 100% of patients, while another study (*n* = 7) reported average diminished protective sensation in the nipple (average SWM score: 3.9).

**Conclusion:**

Our study has shown that objective and patient-reported results of nipple sensitivity support nipple-sparing techniques as a viable option for preserving NAC sensation, although patients can expect a decrease in sensation overall. Neurotization of the NAC during NSM shows promising results of improved postoperative nipple sensitivity, though additional studies are warranted to confirm this finding. Variations between study methodologies highlight the lack of standardization in sensory testing techniques when evaluating NAC sensation.

## 1. Introduction

Breast cancer is the most common type of cancer in women globally, the majority of which require surgery to treat their disease [1]. Although radical mastectomy remains an option, surgical therapy for breast cancer has evolved to include breast-conserving procedures, such as lumpectomies and nipple-sparing mastectomies (NSM), as viable and safe alternatives [2–4]. According to 2016 National Comprehensive Cancer Network (NCCN) guidelines, NSMs are oncologically safe, given specific indications such as early-stage disease, clear nipple margin, and no nipple involvement on imaging [5].

As such, studies have increasingly begun to focus on postmastectomy NAC sensation and how it may affect patient satisfaction after surgery due to improved techniques and patient-reported surgical expectations [6, 7]. NAC sensation is an important factor in maintaining “normality” of the postsurgical breast and plays a major role in women's psychological and sexual health [8]. Several studies report that patients prefer to preserve nipple sensation to achieve a more normal NAC [9, 10]. Although patients can undergo nipple reconstruction, they report lower satisfaction with a reconstructed nipple [11]. Therefore, a greater emphasis has been made to improve NAC sensation after nipple-sparing procedures.

Normal sensation of the breast arises from cutaneous innervation by the intercostal nerves [12]. Medial innervation of the breast is from anterior cutaneous branches of the 1^st^ through 6^th^ intercostal nerves, which produce a medial and lateral branch after passing through the deep fascia at the lateral margin of the sternum [12]. Lateral innervation of the breast arises from lateral cutaneous branches which originate from the 2^nd^ through 7^th^ intercostal nerves at the midaxillary line between the transversus thoracis and internal intercostal muscles [12]. These nerves travel through the external intercostal and serratus anterior muscles, giving off an anterior branch that runs over pectoralis major into the mammary gland where fine branches travel toward the NAC [12].

While current research demonstrates the importance of preserving both the structure and sensory function of the NAC, a succinct review of the current literature on nipple sensitivity after oncologic surgery does not exist. This systematic review and meta-analysis summarizes the current literature on NAC sensation outcomes after nipple-sparing surgeries.

## 2. Methods

For inclusion in this study, all papers included women receiving nipple-sparing surgeries and objective and/or subjective measures of NAC sensitivity. Primary study outcome was the degree of NAC sensation preservation, either by objective sensation exams or patient-reported outcomes (PROs).

### 2.1. Search Strategy

**Figure 1:**
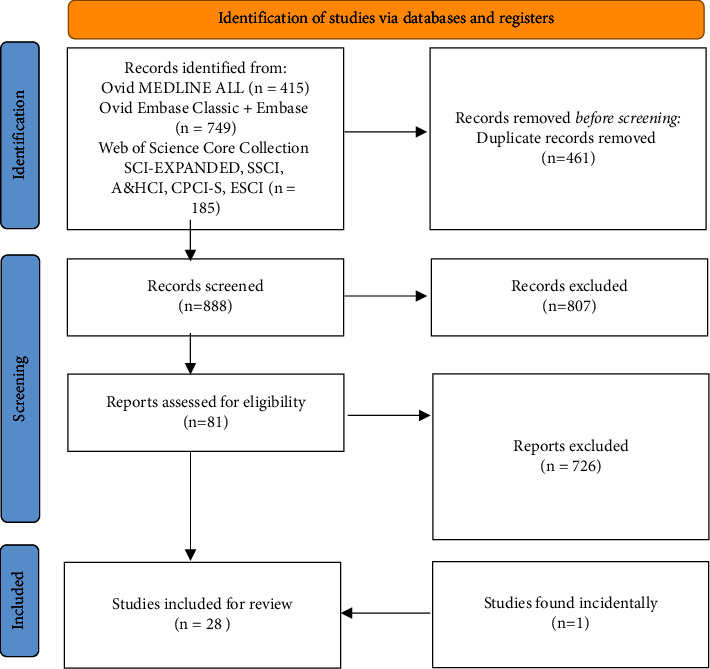
PRISMA flowchart.

The systematic review adhered to the Preferred Reporting Items for Systematic Reviews and Meta-Analyses (PRISMA) guidelines and 2009 checklist as adopted from the Cochrane Collaboration. A systematic search of databases as summarized in Figure 1 was performed using Medical Subject Heading (MeSH) terms and keywords including but not limited to “nipple,” “mastectomy,” “sensation,” and “patient-reported outcome” [13]. Full-text manuscripts available in English and published in peer-reviewed journals were included. No limitations were placed on year of publication or country of origin.

### 2.2. Study Selection

Two independent reviewers screened each citation (V.H. and J.B.) using Rayyan (Qatar Computing Research Institute, Doha, Qatar) systematic review web application. First, studies were screened for relevance based on title and abstract. If a screening decision was not unanimous, a third reviewer (Z.H.) was consulted to discuss their reasoning until consensus was reached. The remaining studies then underwent full-text review. Papers were screened for duplicate patient populations and excluded based on commonalities between author list, study period, and cancer center location.

### 2.3. Data Collection and Analysis

Studies were reviewed to collect primary outcomes and factors that may have impacted these results, such as patient demographics, comorbidities, cancer characteristics, and surgical techniques. PRO measures were evaluated based on total number of patients, and objective sensory testing was evaluated based on total number of operated breasts, nonoperated breasts, and control groups.

Results of investigator-generated surveys were grouped together by reported outcomes. Outcomes such as “excellent” or “good” were recategorized as normal sensation, while “fair” and “poor” were pooled with “decreased” sensation results. We defined “overall maintained” sensation as any sensation above reported absent sensation. Reported outcomes of sensory testing using Semmes−Weinstein monofilaments (SWM) were combined using a modified classification system for assessing quality of sensation as described by Imai et al. (Supplementary Table 1), which correlates higher scores with increased loss of sensation [14]. The continuous variables were analyzed by a random effects model with statistical significance defined as *p* < 0.05.

## 3. Results

### 3.1. Study Selection Process

The initial literature search identified 888 nonduplicate articles. Of these, 81 abstracts were deemed relevant and underwent a full-text review. Fifty-four studies were excluded based on eligibility criteria. One additional study was found incidentally and was added to our analysis. The remaining 28 articles were included for the systematic review, which identified 2915 study subjects (Figure 1).

### 3.2. Study Characteristics

**Figure 2:**
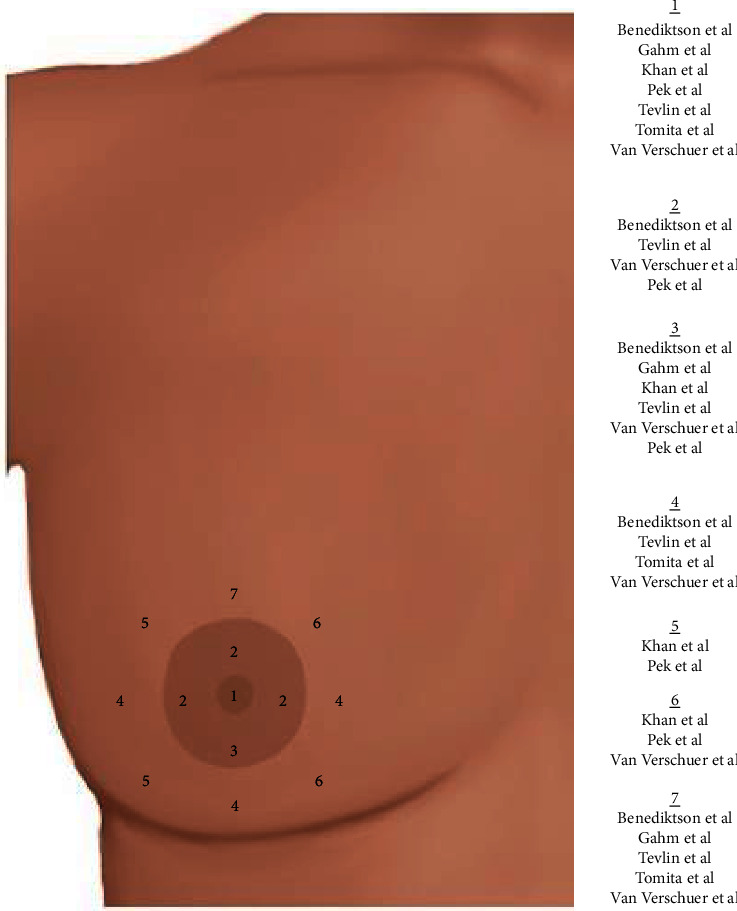
Locations of monofilament sensory testing.

Study characteristics are described in Table1. Of 28 total included studies, twelve (*n* = 578 patients) used objective measures to evaluate sensitivity, such as monofilament testing which included SWM or von Frey hairs [7–10, 15–22]. Areas of the NAC tested with these filaments are displayed in Figure 2. Sixteen studies (*n* = 1,785 patients) assessed patient-reported sensitivity through either validated surveys, such as Breast-Q or investigator-generated surveys [9, 21–35].

### 3.3. Patient Population, Operative Description, and Surgical Outcomes

A total of 2915 patients were identified. Average patient age was 44.3 years (95% CI = 41.4, 47.2), and average body mass index (BMI) was 26.0 kg/m^2^ (95% CI = 23.0, 29.0) (Table 2). Results of the random effects model demonstrated moderate heterogeneity among these study populations, particularly regarding BMI (test of homogeneity, *p*=0.003), though age was comparable between groups (*p*=0.999). 1433 patients received immediate reconstruction compared to 9 patients who received delayed reconstruction. Reconstruction timing was not reported for the remaining 1473 patients.

Upon review of reported surgical techniques, three of the included studies measured NAC sensitivity in patients who received NSM with neurotization procedures (*n* = 203 patients). Tevlin et al. preserved the lateral intercostal nerves during the mastectomy, coapted the nerves to a nerve graft, tunneled them through a free flap, and coapted those nerves to the nerve stumps of the NAC [19]. A similar procedure was performed by Peled and Peled, though the authors specifically reported it using 1 - 2 × 70 mm nerve allografts from Avance (Axogen, Jacksonville, FL) [18]. Peled and Peled additionally used silicone implants and anterior implant coverage with acellular dermal matrices over which the nerve allograft was laid [18]. Djohan et al. also used a technique similar to that of Peled et al. regarding nerve coaptation and the use of cadaveric nerve allografts, but included patients receiving reconstruction with tissue expanders in their study population [36]. These techniques involved dissection of third, fourth, or fifth lateral intercostal nerves at the lateral border of the pectoralis major and coaptation to nerve allografts and remaining nerve stumps of the preserved NAC [18, 19, 36].

### 3.4. Sensory Testing

To measure sensitivity, studies reported both objective and subjective PROMs. Objective sensitivity measures included SWM, von Frey hairs, pinprick sensation tests, and a pressure-specified sensory device. Locations of monofilament testing are shown in Figure 2.

Studies varied with regards to reporting objective sensation in the nipple, areola, or both as part of the NAC. SWM testing (*n* = 138 patients) demonstrated an average loss of protective sensation in the nipple (average SWM score: 4.7) compared to normal or diminished sensation to light touch in nonoperated controls (average SWM score: 2.9, *n* = 195) (Table 3). In the areola, 71 patients reported an overall loss of protective sensation (average SWM score: 5.5) compared to normal or diminished average sensation in nonoperated controls (average SWM score: 3.1, *n* = 57). Three papers utilizing monofilament testing (*n* = 196) demonstrated that 50.7% of patients had preserved NAC sensation when compared to the contralateral breast in unilateral cases or nonoperated controls; however, these studies did not specify the location of sensory testing within the NAC nor provided specific monofilament scores in their results [15, 17, 22].

Other studies reported sensitivity outcomes using the pinprick sensation test, two-point discrimination test, and pressure-specified sensory devices. Chirappapha et al. used the pinprick sensation test and found that 70% (*n* = 7) of patients followed for a year experienced partial sensation recovery [16]. The NAC two-point discrimination test and the pressure-specified sensory device were used by Peled and Djohan, respectively, both of which also performed neurotization and are discussed later in this section. Due to the varied methods of objectively evaluating sensation, their results were not incorporated in our pooled analysis reported above. It was unclear if Stanec et al. (*n* = 288) used patient-reported outcomes or objective sensation measures, but they reported 22% of patients described normal postoperative NAC sensation, 62% reported decreased sensation, and 16% reported no sensation [38].

Of patients who underwent NSM with neurotization, Tevlin et al. (*n* = 7) reported average diminished protective sensation in the nipple (average SWM score: 3.9) and loss of protective sensation in the areola (average SWM score: 4.8) [19]. Peled and Peled (*n* = 16) reported that 87% of patients with minimum 3 months follow-up had intact 2-point discrimination [18]. Djohan et al. assessed sensory preservation using the pressure-specified sensory device and found that patients who underwent NAC neurotization had better sensation in six of eight areas compared with nonneurotized breasts [36].

### 3.5. Patient-Reported Outcomes of NAC Sensation

Patient-reported subjective methods included validated surveys such as the Breast-Q survey (*n* = 102 patients), the Michigan Breast Reconstruction Outcome Study Survey (*n* = 10 patients), and several investigator-generated surveys (*n* = 1643 patients). Investigator-generated surveys assessed patient-reported NAC sensitivity through various scoring systems, most commonly Likert scales. The results showed that of 1565 patients, NAC sensation was “overall maintained” in 29.0% (*n* = 453) of patients, of whom 13.4% (*n* = 61) reported normal sensation and 35.2% (*n* = 132) reported decreased sensation (Table 4). 70.9% of the total patients (*n* = 1110) reported absent sensation (Table 4).

### 3.6. Patient Satisfaction

Many studies reported various aspects of patient satisfaction, including satisfaction overall and with breast aesthetics, nipple appearance, and NAC sensation. Four studies used the Breast-Q survey to assess satisfaction [21, 27, 32, 34]. van Verschuer et al. compared satisfaction between patients receiving skin-sparing mastectomies (SSM) versus NSM. In this cohort, higher Breast-Q scores were reported in the SSM group compared to the NSM group regarding overall satisfaction with breast and surgical outcome. No significant difference existed regarding NAC-specific satisfaction between groups [21]. Kim et al. used an investigator-generated survey rather than Breast-Q to compare these groups; the authors reported no significant difference in breast reconstruction satisfaction between NSM and SSM, yet more NSM patients reported dissatisfaction with nipple position [26]. Manie et al. reported a mean breast satisfaction Breast-Q score of 68.6 (range = 61–74) out of 100, with 100 representing the greatest satisfaction rating [27]. Breast-Q results by Shaffer et al. found that a majority of patients were highly satisfied with their breasts [34]. Manie et al. reported a mean Breast-Q score of 76.4/100 for nipple satisfaction; however, while 89% of patients were satisfied with nipple appearance, only 40% were satisfied with nipple sensation [27, 32]. Studies not utilizing the Breast-Q survey also reported a range of satisfaction with NAC outcomes, including sensation. 17.0% of NSM patients according to Djohan et al. were satisfied with NAC sensation, while the mean satisfaction according to Pek et al. was 2.3 ± 0.7 out of 5 (defining 5 as normal sensation) [9, 31]. Finally, Djohan et al. reported good to excellent satisfaction with nipple aesthetics and sensation in 11 out of 14 breasts [9, 28, 31].

## 4. Discussion

As more women elect to undergo nipple-sparing methods as surgical treatment for their breast cancer, preservation of the NAC and its sensitivity has become more emphasized as a patient-centered outcome [2, 39]. Our study shows that overall NAC sensation was preserved, even though there was average loss of protective sensation when evaluated using objective measures. Results from patient-reported sensitivity measures support these findings and show maintained sensation in almost one-third of patients. Therefore, these results support the increasing success of nipple-sparing procedures as viable options for maintaining nipple sensitivity in surgical treatment of breast cancer.

A 2016 literature review by Sisco and Yao reported similar results regarding sensory outcomes in NSM, particularly that 10–43% of NSM patients self-reported normal sensation [40]. Notably, 14 out of the 28 papers included in our systematic review were published in 2016 or later. Advancements in NAC sensation preservation are expected to have occurred during this time, and continued efforts should be focused on improving neurotization techniques. However, regardless of recent advancements, the findings reported by Sisco and Yao that normal sensation is preserved to varying degrees are supported by our analysis [40].

Three studies included neurotization of the NAC and reported preserved sensation; Peled and Peled demonstrated similar preoperative and postoperative sensation, while Djohan et al. reported decreased sensation in 83% of patients [18, 19, 36]. This difference is likely attributed to variation in the surgical technique, particularly relating to NAC reinnervation. Sensory results reported in Djohan et al. suggest that neurotization of the NAC with cadaveric nerve allografts yields lower-than-expected PROs and satisfaction compared to objective sensory outcomes. In fact, patients in this study reported similar outcomes as other studies without neurotization. Given that Djohan et al. reported a similar surgical technique as Peled, one can postulate that use of tissue expanders, placement of nerve allografts, or type of allograft used may have affected these results, the degree to which each of these technical differences affected the observed outcomes remains unclear. The lower-than-expected sensation preservation with allografts is further supported by Rochlin et al., which performed female-to-male nipple-sparing mastectomy with neurotization. This study did not use allografts and reported no significant difference in NAC sensation between preoperative and postoperative groups compared to a significant decrease in sensation in the nonneurotized control group [41]. However, Ducic et al. report that allografts may in fact be necessary to allow for tensionless repair [42]. Further studies are warranted to overcome the shortcomings with the various neurotization techniques and to assess measures of NAC sensitivity using objective monofilament testing and validated PROMs to determine if these observed outcomes are truly similar.

In addition, Benediktsson et al.mentioned that the increasing difficulty of NAC reinnervation as peripheral nerves is severed [15]. The NAC is innervated by a plexus under the areola formed by variations of the second, third, fourth, and fifth intercostal nerves [43]. These nerves course through the gland to the posterior surface, increasing the likelihood of injury during resection of retroareolar tissue and making the preservation of the anterior branches more important [8, 16]. The first report of sensory repair in autologous breast reconstruction used the anterior ramus of the lateral branch of the fourth intercostal nerve, which emerges at the midaxillary line after traveling through the serratus anterior muscle and later reports used the third anterior intercostal nerve, most likely due to the decreased likelihood of injury [12, 44, 45]. Khan et al. also found that preservation of the anterior intercostal neurovascular bundles resulted in very few reports of severe loss of light touch sensation [17]. Novel techniques, such as the use of endoscopic NSM, may be potentially successful in achieving this preservation [8].

While our study had aimed to analyze data from validated surveys such as Breast-Q and the Michigan Breast Reconstruction Outcome Study Survey, only five included studies utilized one of these surveys [21, 27, 32–34]. Studies using these validated measures generally reported overall patient satisfaction after surgery rather than satisfaction regarding nipple sensation. In contrast, seventeen papers implemented investigator-generated surveys, likely in order to inquire specifically about NAC sensation. This highlights the need for development of a validated breast reconstruction survey that addresses NAC sensation.

Complications such as nipple asymmetry and NAC necrosis were more likely to arise in larger breasts [18, 33]. Although there remains significant potential to improve nipple sensation, many patients reported overall satisfaction with the surgery, despite generally lower satisfaction with nipple sensation. One reason for this may be related to higher patient satisfaction with the aesthetic outcome of preserving the NAC and with the preoccupation of postmastectomy women with disease-free survival as opposed to sensation and arousal [9, 22]. Djohan et al. hypothesized that decreased satisfaction with the procedure may be related to the development of complications, including NAC necrosis, nipple malposition, and delayed wound healing [9, 26]. However, further studies are warranted to assess whether this correlation truly exists.

Several aspects of the included studies may additionally limit the results of this study. The reliance on nonvalidated, investigator-generated surveys may have introduced a reporting bias within our study results. Each investigator-generated survey used different terms to categorize residual NAC sensation after surgery, which would allow the possibility for patients to interpret questions differently, thus affecting the ability to pool results. To mitigate any error due to ambiguity of these study results, we created broadly-defined categories to include the various investigator-generated terms. Differences between individual study questionnaires also reflected the heterogeneity of our included papers, which is another limitation of our study. Of note, variation between study methodologies highlights the lack of standardization in sensory testing techniques when evaluating NAC sensation. Rodriguez−Unda et al. also described the limitations of monofilaments, specifically the need for recalibration with repeated use [7]. Another confounding factor may be the use of different nonoperated control groups in our analysis, which consisted of contralateral nonoperated breasts, preoperative control testing, or patients from a nonoperated control group. It is unclear how inclusion of these control groups may have affected our results. In addition, our findings may be limited due to the inclusion of studies on only women rather than other patient populations such as trans men undergoing female-to-male mastectomies. Finally, the number of studies reporting sensation specific to the NAC following nipple-sparing procedures was limited and demonstrates the need for continued research in this area.

## 5. Conclusion

The literature demonstrates that NAC sensation is preserved in nipple-sparing surgeries alongside overall satisfaction after surgery. Neurotization of the NAC may provide better sensation outcomes with limited improvement in PROs. However, studies on these reinnervation techniques were limited, and additional studies are warranted to confirm this finding. Additionally, future studies should consider creating and utilizing validated patient surveys to allow for more standardized, patient-reported assessments of NAC outcomes. As oncological safety of nipple-sparing procedures has become widely accepted, advancements in NAC sensation preservation have improved patient satisfaction. Despite increasing success in NAC sensation preservation, however, further efforts in this area are needed to improve postoperative NAC sensation and increase patients' quality of life.

## Figures and Tables

**Table 1:** Study characteristics.

Study	Year	Study location	Study design	No. of patients	No. of breasts	Primary surgical technique	Average follow-up period (months)	Measures of sensitivity	Specific measure(s)
Benediktsson et al. [14]	1997	Sweden	Retrospective	80	80	Subcutaneous mastectomy	23.9	Objective sensory exam	Monofilaments
Chirappapha et al. [15]	2018	Thailand	Prospective	52	55	NSM	24	Objective sensory exam	Pinprick sensation test
Djohan et al.[9]	2010	United States	Retrospective	78	116	NSM	50.4	PRO	Investigator-generated survey
Djohan et al. [35]	2020	United States	Prospective	8	15	Not reported	4.18	Objective sensory exam	Pressure-specified sensory device
Eichler et al. [22]	2013	Germany	Retrospective	143	143	Lumpectomy	Not reported	PRO	Investigator-generated survey
Franceschini et al. [23]	2021	Italy	Retrospective	177	299	NSM	16–20*∗*	PRO	Investigator-generated survey
Gahm et al. [21]	2013	Sweden	Prospective	46	92	Risk-reducing mastectomy	29	PRO	Investigator-generated survey, monofilaments
Glaumann et al. [24]	1985	Sweden	Retrospective	72	117	Subcutaneous mastectomy	Not reported	PRO	Investigator-generated survey
Khan et al. [16]	2016	UK	Retrospective	94	181	SSM and NSM	36	Objective sensory exam	Monofilaments
Kim et al. [25]	2019	South Korea	Retrospective	140	140	SSM and NSM	Not reported	PRO	Investigator-generated survey
Manie et al. [26]	2020	Egypt	Prospective	14	Not reported	Batwing mammoplasty	3	PRO	Breast-Q survey
Nahabedian et al. [27]	2006	United States	Retrospective	12	14	Subcutaneous mastectomy	12.9	PRO	Investigator-generated survey
Ou et al. [28]	2015	Taiwan	Retrospective	42	44	NSM	40.9	PRO	Investigator-generated survey
Pan et al. [29]	2019	China	Prospective	41	45	NSM	26	PRO	Investigator-generated survey
Pek et al. [30]	2018	Singapore	Retrospective	133	142	NSM	34.6	PRO	Investigator-generated survey, monofilaments
Peled et al. [31]	2014	United States	Prospective	28	46	NSM	1, 6, and 12*∗*	PRO	Breast-Q survey
Peled et al. [17]	2019	United States		16	31	SSM	3	Objective sensory exam	NAC 2-point discrimination
Petit et al. [32]	2009	Italy	Prospective	972	1001	NSM	20	PRO	Investigator-generated survey
Rodriguez-Unda et al. [7]	2017	United States	Prospective	44	74	NSM	31.6	Objective sensory exam	Pressure-specified sensory device
Shaffer et al. [33]	2019	United States	Retrospective	40	74	NSM	57	PRO	Breast-Q survey
Stanec et al. [36]	2014	Croatia	Retrospective	288	421	Skin and NAC-sparing mastectomy (SNSM)	63	Method not specified	
Tevlin et al. [18]	2020	United States	Retrospective	17	34	NSM	36 (median)*∗*	Objective sensory exam	Monofilaments
Tomita et al. [19]	2011	Japan	Retrospective	67	67	SSM and BCS	31	Objective sensory exam	Monofilaments
van Verschuer et al. [20]	2016	Netherlands	Retrospective	45	89	NSM	27 (median)*∗*	Both	Breast-Q survey, monofilaments
Wang et al. [8]	2017	China	Retrospective	60	60	NSM	35.5	Objective sensory exam	Neuropathy testing pen
Yueh et al. [34]	2009	United States	Retrospective	10	17	NSM	23	PRO	Michigan Breast Reconstruction Outcome Study Survey
Holzgreve et al.	1987	Germany	Not reported	163	302	Subcutaneous mastectomy	Not reported	Method not specified	
Wagner et al. [10]	2012	United States	Prospective	33	54	NSM	15	Objective sensory exam	Time to nipple erection
Total				2915	3753				

BCS, breast-conserving surgery; NAC, nipple-areola complex; NSM, nipple-sparing mastectomy; PRO, patient-reported outcome; SSM, skin-sparing mastectomy. *∗*Reported as a range, median, or individual numbers.

**Table 2:** Patient demographics, disease characteristics, and surgical complications by study.

Study	Patient characteristics				Disease characteristics and treatment					Complications		
	Average age	SD	Average BMI (kg/m^2^)	SD	Breast cancer type	Chemotherapy	Radiation	Reconstruction	Neurotization of NAC	NAC necrosis	Dehiscence	Unplanned reoperation
Benediktsson et al.	54	40–80 (range)^*∗*^	Not provided	Not provided	Not provided	19 (24%)	17 (21%)	Nonautologous = 80 (100%)	0 (0%)	Not provided	Not provided	Not provided

Chirappapha et al.	43	30–60 (range)^*∗*^	23.1	16.3–43.8 (range)^*∗*^	Invasive carcinoma = 33 (77%) DCIS = 10 (23%) Benign phyllodes tumor = 3 (6%) Benign condition = 8 (15%)	Not provided	Not provided	Both autologous and nonautologous (numbers not specified)	0 (0%)	11 (20%)	Not provided	3 (5.5%)

Djohan et al.	46.5	9.42	24	4.79	Prophylactic = 12 (15.4%) DCIS (29, 37.2%) LCIS (3, 3.8%) Infiltrating (34, 43.5%)	Not provided	Not provided	Nonautologous, *n* = 84 (72.4%) Autologous, *n* = 32 (27.6%)	0 (0%)	2 (2.6%)	Not provided	31 (39.7%)

Djohan et al.	38.12	7.5	23.66 (4.19)	4.19	Not provided	4 (26.8%)	0 (0%)	Nonautologous, *n* = 15 (100%)	15 (100%)	2 (13.4%)	1 (6.7%)	Not provided

Eichler et al.	52.9	9.3	Not provided	Not provided	DCIS (14, 9.8%) IDC (104, 72.8%) ILC (18, 12.6%) Others (6, 4.2%)	94 (65.8%)	Not provided	Not provided	0 (0%)	Not provided	Not provided	Not provided

Franceschini et al.	45.3	27–73 (range)^*∗*^	24.4	17.5–29.4 (range)^*∗*^	Not provided	73 (41.2%)	45 (25.4%)	Nonautologous = 177 (100%)	0 (0%)	1 (1.2%)	Not provided	Not provided

Gahm et al.	39 (median)*∗∗*	26–58 (range)*∗*	Not provided	Not provided	Prophylactic = 46 (100%)	0	Not provided	Nonautologous = 46 (100%)	0 (0%)	Not provided	Not provided	Not provided

Glaumann et al.	Not provided	Not provided	Not provided	Not provided	Not provided	Not provided	Not provided	Autologous = 7, 9.7% Nonautologous = 62, 86.1%	0 (0%)	4 (3.4%)	Not provided	41 (35.0%)

Khan et al.	51 (median)^*∗∗*^	30–72 (range)^*∗*^	Not provided	Not provided	Not provided	Not provided	Not provided	Autologous = 4 (2.2%) Nonautologous = 73 (40.3%) Both = 68 (37.6%)	0 (0%)	Not provided	Not provided	Not provided

Kim et al.	44.5	7.53	21.3	2.52	Not provided	64 (45.7%)	Not provided	Nonautologous = 140 (100%)	0 (0%)	Not provided	Not provided	5 (3.6%)

Manie et al.	50	41–66 (range)^*∗*^	36.7	31.6–44.9 (range)^*∗*^	Not provided	5 (35.7%)	Not provided	Not provided	0 (0%)	0 (0%)	1 (7%)	1 (7%)

Nahabedian et al.	48.8	35–72 (range)^*∗*^	Not provided	Not provided	Not provided	Not provided	Not provided	Nonautologous = 8 (57.1%) Autologous = 6 (42.8%)	0 (0%)	1 (7.1%)	Not provided	5 (35.7%)

Ou et al.	45.2	29–67 (range)^*∗*^	21.8	17.48–27.15 (range)^*∗*^	DCIS (12, 27.3%) LCIS (1, 2.3%) IDC (29, 65.9%) ILC (1, 2.3%) Phyllodes (1, 2.3%)	21 (47.7%)	7 (15.9%)	Nonautologous = 38 (86.4%) Autologous = 6 (13.6%)	0 (0%)	6 (13.6%)	Not provided	4 (9.1%)

Pan et al.	41 (median)^*∗∗*^	35–45 (IQR)^*∗∗*^	Not provided	Not provided	IDC (27, 65.9%)	0 (0%)	45 (100%)	Nonautologous = 41 (100%)^*∗∗∗*^	0 (0%)	3 (7.3%)	Not provided	Not provided

Pek et al.	47	8.8	23.5	4.5	Not provided	Not provided	0 (0%)	Autologous = 148 (84.6%) Nonautologous = 27 (15.4%)	0 (0%)	17 (12.0%)	Not provided	8 (5.6%)

Peled et al.	47.8	30.4–69.9 (range)^*∗*^	23.2	17.3–27.7 (range)^*∗*^	Not provided	14 (50%)	4 (14.3%)	Nonautologous = 46 (100%)	0 (0%)	3 (10.7%)	Not provided	3 (10.7%)

Peled et al.	Not provided	Not provided	Not provided	Not provided	Not provided	Not provided	Not provided	Nonautologous = 31 (100%)	31 (100%)	Not provided	Not provided	Not provided

Petit et al.	46	20–73 (range)^*∗*^	Not provided	Not provided	Invasive carcinoma (819, 82%) IDC (182, 18.2%)	Not provided	1001 (100%)	Nonautologous = 991 (99%) Autologous = 10 (1%)	0 (0%)	80 (8.0%)	Not provided	93 (9.3%)

Rodriguez-Unda et al.	50.2	8.6	25.6	4.1	Not provided	35 (79.5%)	16 (36.4%)	Nonautologous = 37 (50%) Autologous = 37 (50%)	0 (0%)	Not provided	Not provided	108 (245%)

Shaffer et al.	48	29–63 (range)^*∗*^	Not provided	Not provided	Atypia/DCIS (2, 5%) Invasive cancer (20, 50%)	12 (30%)	3, 7.5%	Nonautologous = 74 (100%)	0 (0%)	0 (0%)	Not provided	Not provided

Stanec et al.	46.6	9	Not provided	Not provided	IDC (212, 50.4%) ILC (71, 16.9%) DCIS (63, 15%) Others (28, 6.5%)	Not provided	Not provided	Autologous (276, 65.6%) Nonautologous (145, 34.4%)	0 (0%)	29 (10.1%)	Not provided	Not provided

Tevlin et al.	49	10.4	28.7	4.8	Not provided	Not provided	0, 0%	Autologous (14, 100%)	14 (100%)	Not provided	Not provided	Not provided

Tomita et al.	46	26–66 (range)^*∗*^	Not provided	Not provided	Not provided	32 (30.8%)	70 (67.3%)	Autologous = 104 (100%)	0 (0%)	Not provided	Not provided	Not provided

van Verschuer et al.	40 (median)^*∗∗*^	26–71 (range)^*∗*^	Not provided	Not provided	Not provided	5 (11.1%)	2 (4.4%)	Nonautologous = 45 (100%)	0 (0%)	7 (15.6%)	Not provided	17 (37.8%)

Wang et al.	44.22	8.56	Not provided	Not provided	Not provided	54 (83.3%)	27 (45.0%)	Autologous = 19 (31.7%) Nonautologous = 37 (61.7%) None = 4 (6.6%)	0 (0%)	Not provided	Not provided	Not provided

Yueh et al.	44	25–57 (range)^*∗*^	Not provided	Not provided	Benign breast disease (3, 17.6%) LCIS (2, 11.8%) DCIS (1, 5.9%) Invasive carcinoma (1, 5.9%)	Not provided	Not provided	Nonautologous = 15 (88.3%) Autologous = 2 (11.7%)	0 (0%)	3 (17.6%)	Not provided	7 (41.1%)

^*∗*^Data reported as a range. ^*∗∗*^Data reported as a median and/or interquartile range (IQR). ^*∗∗∗*^Includes delayed reconstruction (22%). All other studies include only immediate reconstruction. DCIS, ductal carcinoma in situ; IDC, invasive ductal carcinoma; ILC, invasive lobular carcinoma; IQR, interquartile range; LCIS, lobular carcinoma in situ; NAC, nipple-areola complex; SD, standard deviation.

**Table 3:** Objective outcomes and sensory testing.

Location	Population	Studies reported	Studies reported	No. of breasts	Average SWM value	Total breasts	Weighted average SWM value	Sensory perception
Nipple	NSM	4	Tevlin (NSM)	20	4.9	138	4.7	Loss of protective sensation
			Pek	15	4.435			
			Tomita (BCS)	47	3.84			
			Tomita (SSM)	20	4.56			
			van Verschuer	36	5.814			
	nNSM	1	Tevlin (nNSM)	14	3.9	14	3.9	Diminished light touch to diminished protective sensation
	Non-op control	4	Tevlin (nNSM control)	14	3.2	195	2.9	Normal to diminished light touch
			Tevlin (NSM control)	20	2.83			
			Pek	15	3.142			
			Tomita	104	2.83			
			van Verschuer	42	2.908			

Areola	NSM	3	Tevlin (NSM)	20	5.68	71	5.5	Loss of protective sensation
			Pek	15	4.41			
			van Verschuer	36	5.76			
	nNSM	1	Tevlin (nNSM)	14	4.84	14	4.8	Loss of protective sensation
	Non-op control	2	Pek	15	2.908	57	3.1	Normal to diminished light touch
			van Verschuer	42	3.123			

BCS, breast-conserving surgery; NSM, nipple-sparing mastectomy; nNSM, nipple-sparing mastectomy with neurotization of nipple-areola complex; non-op, not operated; SSM, skin-sparing mastectomy; SWM, Semmes–Weinstein monofilaments.

**Table 4:** Patient reported outcomes.

	No. of patients	No. of patients reporting level of sensation					
		Normal	Maintained	Decreased	Poor	Overall maintained	Absent
Eichler et al.	143	22	.	28	.	50	93
Franceschini et al.	177	.	69	.	.	69	108
Gahm et al.	46	0	.	29	.	29	17
Glaumann et al.	72	12	.	35	.	47	25
Nahabedian et al.	12	.	.	5	.	5	7
Ou et al.	42	24	.	7	9	41	.
Pan et al.	41	0	.	3	29	32	9
Petit et al.	972	.	146	.	.	146	826
Shaffer et al.	40	.	2	7	5	14	26
van Verschuer et al.	20	2	.	18	.	20	.
	No. of patients reporting sensation	61	217	132	43	453	1110
	Total patients	364	1149	376	123	1565	1565
	Overall (%)	16.6%	18.9%	35.2%	34.9%	29.0%	70.9%

## Data Availability

The data supporting this systematic review and meta-analysis are from previously reported studies and datasets, which have been cited. The processed data are available from the corresponding author upon request.

## Supplementary Materials

Supplementary Materials Imai et al. correlated the quality of sensation with the monofilament markings and their calculated forces. This study adapted those classifications when assessing objective sensory outcomes in order to evaluate the results in a standardized manner.
