# Supplementary material for: Preserving Nipple Sensitivity after Breast Cancer Surgery: A Systematic Review and Meta-Analysis
Source: Breast J. 2022 Nov 19;2022:9654741. doi: 10.1155/2022/9654741 (PMC9701124; doi:10.1155/2022/9654741)
Supplement: Supplementary Materials — Imai et al. correlated the quality of sensation with the monofilament markings and their calculated forces. This study adapted those classifications when assessing objective sensory outcomes in order to evaluate the results in a standardized manner. [file 9654741.f1.docx]

**Supplementary Description**

Imai et al correlated the quality of sensation with the monofilament markings and their calculated forces. This study adapted those classifications when assessing objective sensory outcomes in order to evaluate the results in a standardized manner.

**Supplementary Table 1: Classification of the Quality of Sensation based on Monofilament Testing***

| Quality of Sensation | Filament Marking | Calculated Force (grams) |
| --- | --- | --- |
| Normal | 1.65 – 2.83 | 0.0045 – 0.068 |
| Diminished light touch | 3.22 – 3.61 | 0.166 – 0.408 |
| Diminished protective sensation | 3.84 – 4.31 | 0.697 – 2.06 |
| Loss of protective sensation | 4.56 – 6.65 | 3.63 - 447 |
| > 6.65 | > 447 |  |
| *adapted from Imai et al.^13^ | | |
